# Lateral habenula-rostromedial tegmental nucleus circuit mediates inflammatory pain in mice

**DOI:** 10.1186/s10194-025-02052-w

**Published:** 2025-05-06

**Authors:** Yanfei Sun, Jing Cao, Chunpeng Xu, Jiangtao Sun, Xiaofeng Liu, Zhenguang Shi, SiMeng An, Danyang Zhao, Dongjie Sun, Xuxin Wang, Guoyan Zhao, Chi Zhang, Guangjian Li, Jinyu Xiao, Jing Yang, Hua Zhao

**Affiliations:** 1 School of Life Sciences, Changchun Normal University, Changchun, 130021 China; 2 Department of Physiology, College of Basic Medical Sciences, Jilin University, Changchun, 130021 China; 3 China-Japan Union Hospital of Jilin University, Changchun, 130033 China; 4 Van-Research Intelligence Technology Co., Ltd., Zhengzhou, 450007 China; 5 Neuroscience Research Center, First Hospital of Jilin University, Changchun, 130021 China

**Keywords:** Lateral habenula, Rostromedial tegmental nucleus, Dorsal raphe nucleus, Glutamatergic neurons, Pain, Neural pathway

## Abstract

**Background:**

The monoamine system, particularly the serotonergic neurons in the dorsal raphe nucleus (DRN), associated with the synthesis and release of 5-hydroxytryptamine, is crucial for regulating pain. The lateral habenula (LHb) modulates DRN neurons by acting through GABAergic neurons located in the rostromedial tegmental nucleus (RMTg). However, the role of RMTg in mediating the LHb and regulating pain remains unclear. Thus, we aimed to assess the role of the LHb-RMTg pathway in inflammatory pain.

**Methods:**

Male C57BL/6 mice were used in the chemogenetic experiments, while male and female Vglut2-ires-cre mice were used in the optogenetic experiments; in both experiments, inflammatory pain model and control groups were established. We performed the Hargreaves and Von Frey tests to assess nociceptive behavior as well as immunohistochemistry staining after chemogenetic activation experiments. Statistical analyses were performed using a *t-*test, one-way analysis of variance (normally distributed data) or Kruskal–Wallis test (non-normally distributed data) and two-way analysis of variance.

**Results:**

Chemogenetic activation/inhibition of RMTg-projecting LHb excitatory neurons was sufficient to decrease or increase heat sensitivity thresholds. Additionally, inhibition of the LHb-RMTg circuit reversed the decreased heat sensitivity thresholds under inflammatory pain conditions using chemogenetic and optogenetic approaches. However, this circuit did not affect mechanical allodynia thresholds, and chemogenetic activation of the circuit decreased c-Fos immunoreactivity in the DRN.

**Conclusions:**

Our results indicate that activating glutamatergic neurons within the LHb heightens pain sensitivity by triggering GABAergic neurons in the RMTg, which in turn influences neuronal activity in the DRN. This research offers fresh perspectives on the pain mechanism, potentially revealing new therapeutic avenues for managing inflammatory pain.

**Graphical Abstract:**

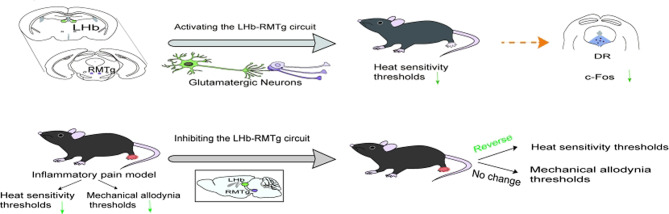

**Supplementary Information:**

The online version contains supplementary material available at 10.1186/s10194-025-02052-w.

## Background

Inflammatory pain affects the quality of life and remains poorly understood, especially the brain circuitry involved. The lateral habenula (LHb), which is primarily made of glutamatergic neurons, relays signals between the limbic forebrain and midbrain structures, participating in numerous biological processes and behaviors, such as pain regulation [1–5]. Early studies found that LHb neuron firing rates and expression of the c-Fos increase in response to painful stimulation [6, 7]. Meanwhile, manipulating LHb activity with lidocaine results in antinociception [8]. Likewise, recent research indicates that LHb neurons exhibit heightened activity following sciatic nerve injury, and targeted inhibition of LHb glutamatergic neurons alleviates mechanical pain [9]. These results suggest a role for LHb in pain modulation.

Anatomically, LHb neurons project extensively to the pain-regulating nuclei within the periaqueductal gray and dorsal raphe nucleus (DRN) [10, 11]. Stimulation of the DRN and manipulation of 5-hydroxytryptamine (5-HT) neurons in the DRN result in potent antinociceptive effects [12–14]. The LHb regulates the output of 5-HT neurons in the DRN. Electrical stimulation of the LHb markedly suppresses the firing activity of DRN neurons [15], and increased LHb and decreased DRN neuronal activity have been observed in pathological rat pain models [10]. These studies suggest that LHb modulates nociception via its influence on the DRN.

The rostromedial tegmental nucleus (RMTg), a recently defined GABAergic brain region, receives glutamatergic input from LHb, which is projected onto the DRN [16]. RMTg regulates reward prediction signals and aversion related to LHb [17]. A recent chemogenetic investigation demonstrated that targeted activation and suppression of RMTg GABAergic neurons resulted in nociception and analgesia, respectively [18], thereby suggesting that RMTg receives glutamatergic input from LHb and participates in pain regulation. Moreover, the LHb-RMTg pathway regulates emotion- and stress-induced sleep alterations [19–21] and affects pain perception. Therefore, the LHb-RMTg pathway may be involved in pain regulation, and further research is needed to understand how it works and how it may be leveraged in the clinic.

In this study, we aimed to employ optogenetic methods and chemogenetic approaches using Designer Receptors Exclusively Activated by Designer Drugs (DREADD) alongside behavioral techniques to modulate excitatory RMTg-projecting LHb neurons, and to evaluate the role of the LHb-RMTg pathway in inflammatory pain. Additionally, we used immunohistochemistry to determine the underlying molecular mechanism.

## Methods

Male C57BL/6 mice were used for chemogenetic experiments. Vglut2-ires-cre mice of both sexes were used for the optogenetic experiments. The mice were provided by the Shanghai Institute for Biological Sciences, Chinese Academy of Sciences. All mice were kept in regulated environments with a 12-hour light-dark cycle, with unrestricted access to food and water. Animal use in this study was approved by the Animal Care Research Committee of Jilin University, following established guidelines for experimental animal care and usage. Mice, aged between 6 and 8 weeks, were group-housed, with no significant differences observed between sexes.

### Experimental protocol and groups

**Fig. 1:**
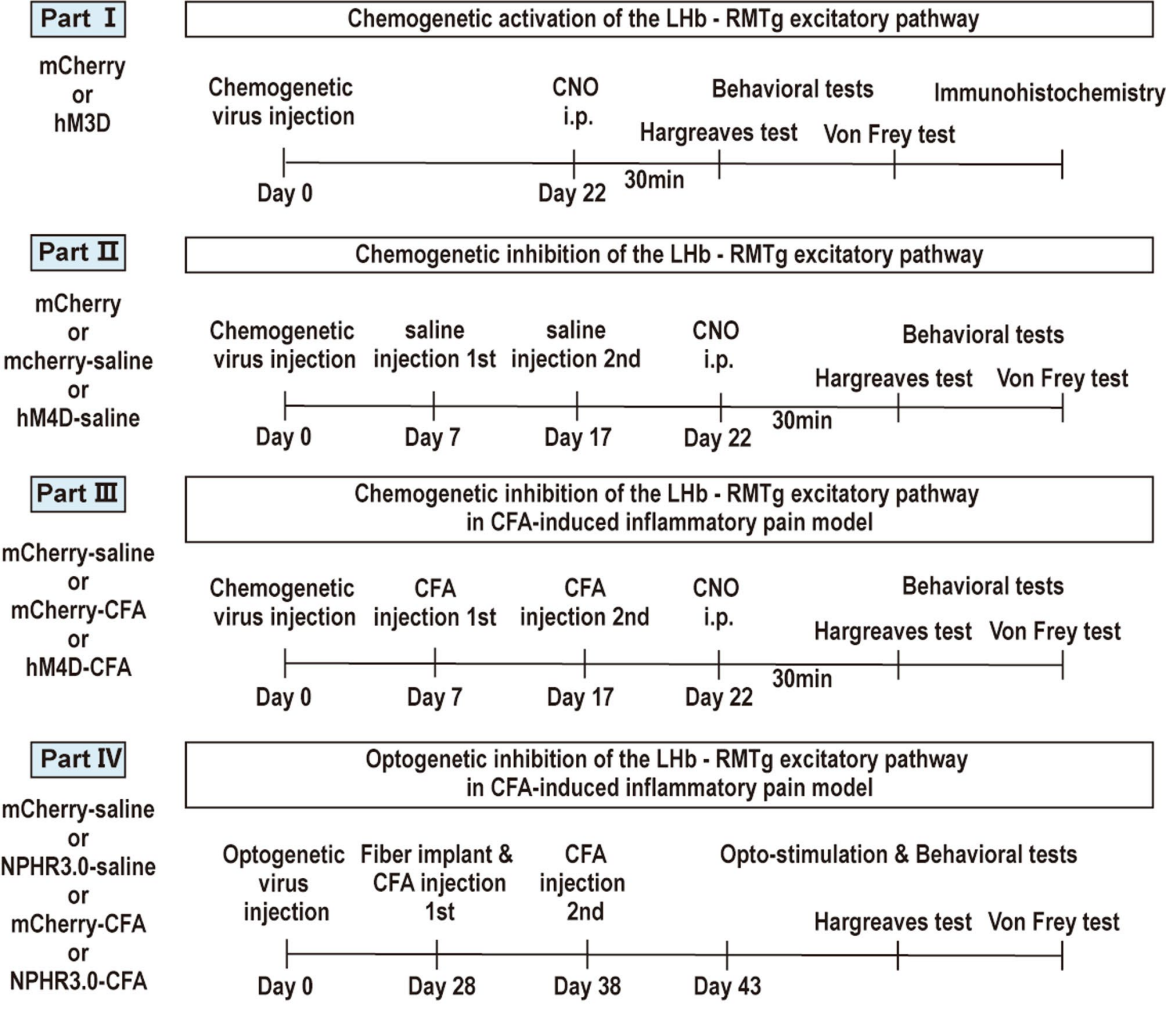
Experimental protocol and groups

The experiment was divided into four parts (Fig. 1). Chemogenetic approaches were used to modulate excitatory RMTg-projecting LHb neurons, and to evaluate the role of the LHb-RMTg pathway in pain behaviors. Optogenetic methods were used to modulate the instantaneous regulation of pain associated with glutamatergic terminals from LHb to RMTg.

First, the chemogenetic activation of the LHb-RMTg pathway was performed. This part was divided into hM3D and mCherry groups. After viral infection, Clozapine-N-oxide (CNO) was administered, and 30 min later, Hargreaves and Von Frey tests were conducted. Following the behavioral tests, c-Fos immunoreactivity in the DRN was performed to assess neuronal activity.

Second, we aimed to investigate the effects of Hargreaves and Von Frey tests after the chemogenetic inhibition of the LHb-RMTg pathway. This part was divided into mCherry, mCherry-saline and hM4D-saline groups.

Third, we examined the influence of chemogenetic inhibition of the LHb-RMTg pathway on pain behavior in the complete freund’s adjuvant (CFA) induced inflammatory pain model. This part was divided into hM4D-CFA, mCherry-CFA and mCherry-saline groups.

Fourth, we studied the influence of optogenetic inhibition of the terminals from LHb to RMTg on pain behavior in the pain model. This part was divided into NPHR3.0-saline, mCherry-saline, NPHR3.0-CFA and mCherry-CFA groups.

### Inflammatory pain model

The experimental mice were prepared by injecting the left hind paw plantar surface with 10 µL CFA (Sigma-Aldrich), and a second injection was administered 10 days later to ensure persistent pain [22]. Saline (0.9% NaCl) was used as the control.

### Virus preparation and stereotactic surgery

Stereotaxic intracranial viral injections were administered to mice anesthetized using 1% pentobarbital (80 mg/kg). Afterward, the mice were positioned on a stereotaxic device (RWD, Shenzhen, China). The virus was delivered to each animal via a microinjection pump (LSP02-1B, Shenzhen, China) using a glass electrode securely affixed to the needle of the microinjector. To specifically infect RMTg-projecting LHb neurons in chemogenetic experiments, 150 nL of adeno-associated virus (AAV)2/Retro-CaMKIIa-Cre (Cat#PT-0220) was injected into RMTg and 200 nL of AAV2/9-DIO-hM3D-mCherry (Cat#PT-0042), AAV2/9-DIO-hM4D-mCherry (Cat#PT-0043) or AAV2/9-DIO-mCherry (Cat#PT-0013) into LHb in C57BL/6 mice bilaterally. For optogenetic studies, 200 nL of AAV2/9-DIO-eNpHR3.0-mCherry (Cat# PT-0007) or AAV2/9-DIO-mCherry (Cat# PT-0013) was infused into LHb in Vglut2-ires-cre mice to stimulate the terminals of LHb in RMTg. Post-injection, the glass electrode was kept in place for 10 min before removal. For optogenetic stimulation, optic fibers were implanted above RMTg bilaterally. Stereotaxic coordinates were determined according to the mouse brain atlas by Paxinos and Franklin (2001). The coordinates relative to the bregma for LHb were anteroposterior (AP), − 1.6 mm; mediolateral (ML), ± 0.4 mm; dorsoventral (DV), − 2.7 mm; for RMTg: AP, − 4.16 mm; ML, ± 0.42 mm; DV, − 4.4 mm; for optic fibers above RMTg: AP, − 4.16 mm; ML, ± 1.62 mm; DV, − 4.3 mm, at 15°. For the procedure, all AAVs were purchased from Brain VTA Technology Co., Ltd. (Wuhan, China) and optic fibers (Ferrule outer diameter: 1.25 mm; Fiber core: 200 μm; NA: 0.37) from Inper Technology Co., Ltd. (Hangzhou, China).

### Chemogenetic and optogenetic manipulation

Behavioral tests for chemogenetic experiments were conducted 3 weeks later. Prior to behavioral tests or sacrifice, the mice were administered intraperitoneal injections of CNO (0.33 mg/mL, APExBIO, A3317) or saline 30 min in advance. The optogenetic behavioral tests were performed after 6 weeks. A paradigm of 589 nm light laser (Aurora-220, NEW DOON, China) was used (5-ms pulses at 60 Hz, 4–8 mW) bilaterally and light output through the optical fiber was checked using a fiber optic power meter (PM20A, Thorlabs, USA).

### Nociceptive behavioral tests

We performed the Hargreaves and Von Frey tests to assess nociceptive behavior. The Hargreaves test measured the paw withdrawal thermal latency (PWTL) in response to a constant-intensity radiant heat source (PL-200, China) on the left hind paw to evaluate heat sensitivity thresholds. The obtained values were averaged after 3–5 trials following 30 min of habituation [18]. The von Frey test was employed to assess mechanical allodynia specifically measuring the paw withdrawal mechanical threshold (PWMT) using the up–down method with von Frey filaments (0.008, 0.04, 0.07, 0.16, 0.4, 0.6, 1.0, 1.4, and 2.0 g). Before testing, mice were placed in a plastic cage with a wire mesh bottom for 30 min to acclimate. Each von Frey filament was applied perpendicularly to the mid-plantar surface of the left hind paw for 4 s at 30-s intervals, and the 50% response threshold was calculated [23, 24].

### Immunohistochemistry

After chemogenetic activation experiments, c-Fos^+^ expression in the DRN was examined. The mice underwent intraperitoneal injections of CNO, followed by harvesting of brains 30 min later. These brains were then immersed in 4% paraformaldehyde for 24 h to ensure proper fixation. These tissues were sliced into 3-µm paraffinized brain sections with a microtome (RM2245, Leica, Germany). The sections were prepared for immunohistochemical staining with rabbit anti-c-Fos antibody (1:500; Abcam, Cambridge, UK; ab190289). Subsequently, the sections were observed using an OLYMPUS microscope (IX71, Japan), and c-Fos–immunopositive neurons were counted using the Image Pro-Plus software.

### Injection site verification

In order to evaluate the effectiveness of the viral injection, the brains of mice were perfused and fixed with 4% paraformaldehyde. Subsequently, the brains were dehydrated using a graded series of 10%, 20%, and 30% sucrose solutions (Macklin, China), sliced using a freezing microtome (CM1950, Leica, Germany), and inspected with a fluorescence microscope (IX71, OLYMPUS, Japan) to identify the accuracy of viral injections. To assess whether the virus injection can effectively target the RMTg, we co-injected of AAV2/Retro-CaMKIIα-Cre and Alexa Fluor 488-conjugated cholera toxin subunit B (CTB-488) as a green fluorescent/tag into the RMTg. Subsequent histological images were inspected.

### Data analysis and statistics

Data are presented as mean ± standard error. The Shapiro–Wilk test was used to check the normality of variable distribution before analysis. The t-test or one-way ANOVA was applied to normally distributed variables. Non-normally distributed variables were analyzed using the Kruskal–Wallis test. Post-hoc comparisons were performed using either Tukey’s or Dunnett’s test. Two-way ANOVA was used followed by Bonferroni’s multiple-comparison post hoc tests. GraphPad Prism version 8.0 software was used for analysis, and statistical significance was defined as *p* < 0.05.

## Results

### Behavioral testing for pain and c-Fos expression in DRN following activating the LHb-RMTg excitatory pathway

**Fig. 2:**
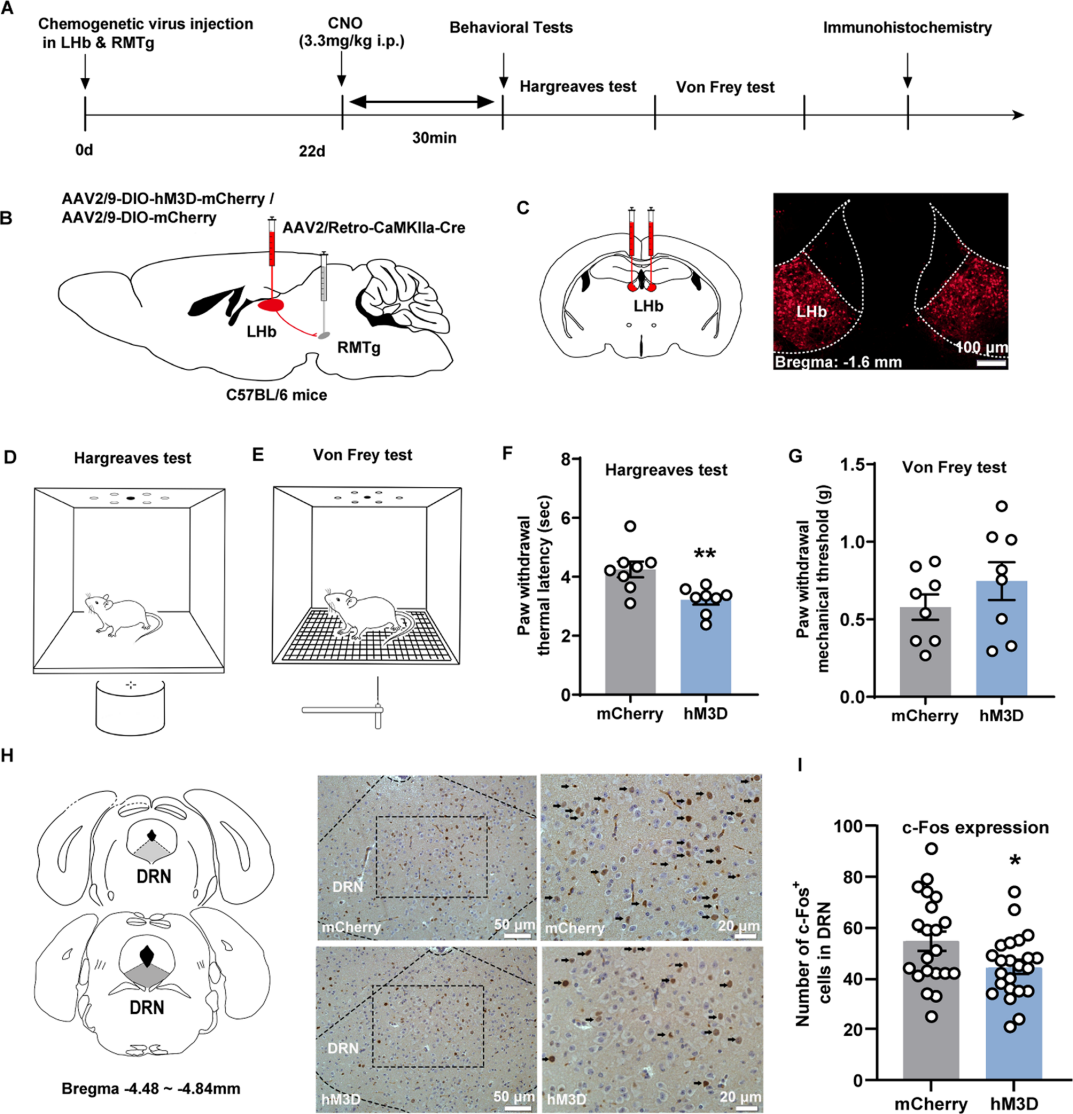
Behavioral testing for pain and c-Fos expression in DRN following activating the LHb-RMTg excitatory pathway. (**A**) Flow diagram of the experiment for DREADD-mediated activation of the LHb-RMTg pathway. (**B**) Schematics: AAV2/9-DIO-hM3D-mCherry or AAV2/9-DIO-mCherry is microinjected into LHb, whereas AAV2/Retro-CaMKIIa-Cre is microinjected into RMTg. (**C**) Representative fluorescence images: AAV2/9-DIO-hM3D-mCherry expressed in LHb (Scale bar: 100 μm). (**D**, **F**) Schematics and corresponding graphs illustrating the Hargreaves test performed in the chemogenetic experiment. (**E**, **G**) Schematics and corresponding graphs illustrating the Von Frey test performed in the chemogenetic experiment. (**H**) Representative images of c-Fos immunolabeled cells in the DRN of the mCherry and hM3D groups (black arrows indicate c-Fos positive cells; scale bars: left panels, 50 μm; right panels, 20 μm). (**I**) Quantitative analysis of c-Fos positive cells in the DRN. Mean ± standard error of mean values are presented; *p < 0.05, **p < 0.01. DREADD, Designer Receptors Exclusively Activated by Designer Drug; DRN, dorsal raphe nucleus; LHb, lateral habenula; RMTg, rostromedial tegmental nucleus

The experimental procedure for activating the LHb-RMTg excitatory pathway is depicted in Fig. 2A. To activate RMTg-projecting LHb excitatory neurons using chemogenetics, AAV2/9-DIO-hM3D-mCherry or AAV2/9-DIO-mCherry as a control was injected into LHb, whereas AAV2/Retro-CaMKIIa-Cre was injected into RMTg and schematics are shown in Fig. 2B. Viral expression in the LHb was shown in Fig. 2C and specific targeting of the RMTg was validated with no off-target signals in adjacent regions (Figure S1A, Supplementary Materials). Meanwhile Fig. 2D and E show schematic representations of the Hargreaves and Von Frey tests, respectively. In the Hargreaves test, following chemogenetic activation of the LHb-RMTg excitatory pathway, the PWTL was recorded as 3.22 ± 0.16 and 4.25 ± 0.27 s for the hM3D and mCherry groups, respectively. Notably, there was a significant decrease in the average PWTL of the hM3D group compared with that of the controls (Fig. 2F, *p* = 0.0051, t = 3.32, *n* = 8 for mCherry group, *n* = 8 for hM3D group, unpaired *t*-test). In contrast, during the Von Frey test, the PWMT of hM3D group was 0.75 ± 0.12 g. In comparison, the mCherry group had a threshold of 0.58 ± 0.082 g. This result demonstrates that activation of the LHb-RMTg pathway does not significantly influence PWMT (Fig. 2G, *p* = 0.27, t = 1.14, *n* = 8 for mCherry group, *n* = 8 for hM3D group, unpaired *t*-test). Therefore, DREADD-mediated activation of the LHb-RMTg circuit led to increased heat sensitivity, with no impact on the mechanical threshold.

c-Fos expression in the DRN following DREADD-mediated activation of the LHb-RMTg excitatory pathway was detected. Representative diagrams of DRN and c-Fos-immunolabelled cells were shown in Fig. 2H. The hM3D group exhibited a significant reduction in the count of c-Fos-labeled cells compared with the mCherry group (Fig. 2I, *p* = 0.033, t = 2.2, *n* = 21 sections from 4 mice for mCherry group, *n* = 23 sections from 4 mice for hM3D group, unpaired *t*-test).

### Behavioral testing for pain following inhibiting the LHb-RMTg excitatory pathway

**Fig. 3:**
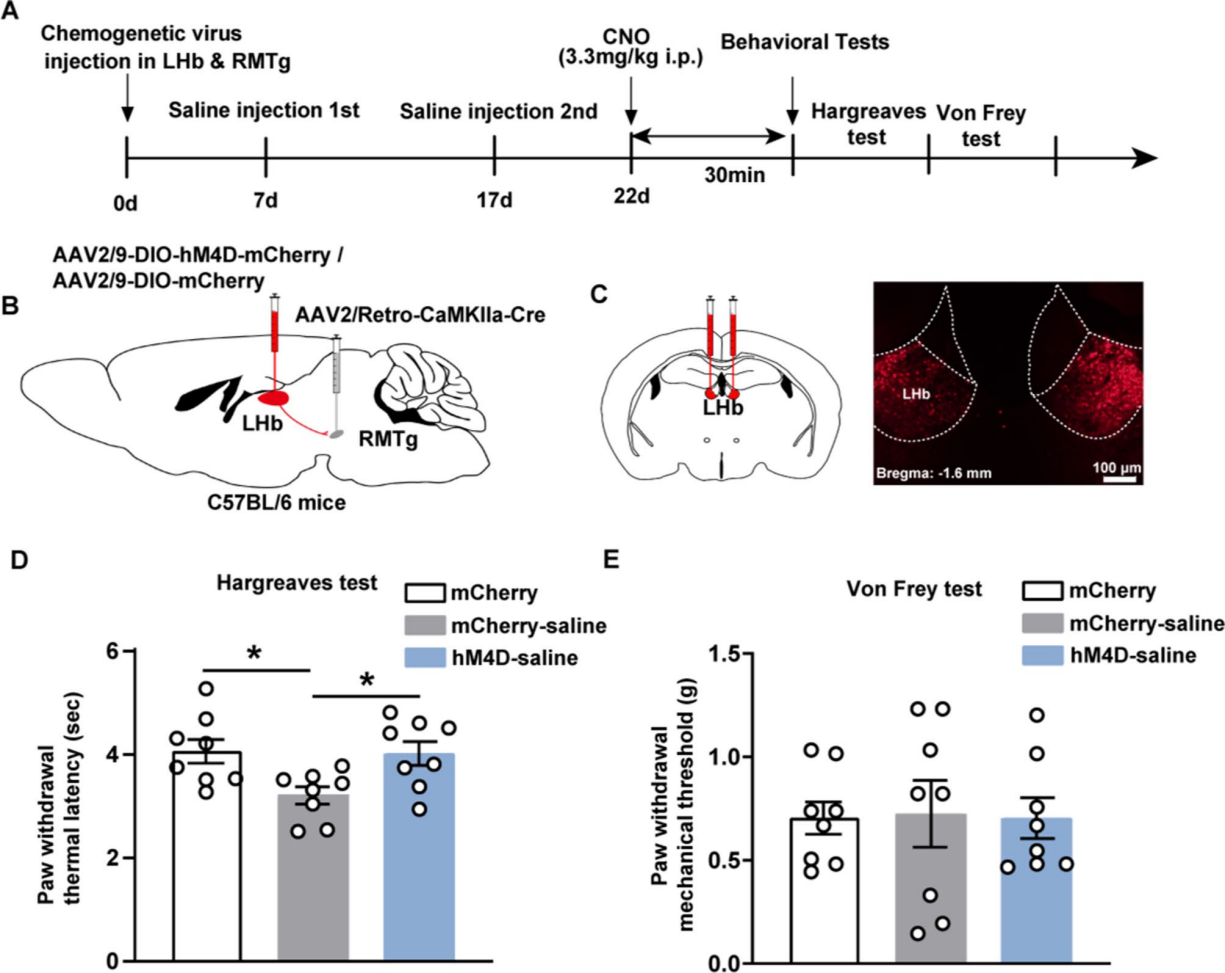
Behavioral testing for pain following inhibiting the LHb-RMTg excitatory pathway. (**A**) Flow diagram of the experiment for DREADD-mediated inhibition of the LHb-RMTg pathway. (**B**) Schematics: AAV2/9-DIO-hM4D-mCherry or AAV2/9-DIO-mCherry is microinjected into LHb, whereas AAV2/Retro-CaMKIIa-Cre is microinjected into RMTg. (**C**) Representative fluorescence images: AAV2/9-DIO-hM4D-mCherry expressed in LHb (Scale bar: 100 μm). (**D**) Graphs of the Hargreaves test in the chemogenetic experiment. (**E**) Graphs of the Von Frey test in the chemogenetic experiment. Mean ± standard error of mean values are presented; *p < 0.05. DREADD, Designer Receptors Exclusively Activated by Designer Drug; LHb, lateral habenula; RMTg, rostromedial tegmental nucleus

We investigated the effects of DREADD-mediated inhibition of this neural circuit to elucidate the impact of RMTg-projecting LHb neurons on pain perception. The experimental design is depicted in Fig. 3A. Specifically, AAV2/9-DIO-hM4D-mCherry or AAV2/9-DIO-mCherry serving as the control was delivered into LHb, while AAV2/Retro-CaMKIIa-Cre was distinctly delivered into RMTg. Schematics are shown in Fig. 3B. Viral expression in the LHb was shown in Fig. 3C, while the validation of viral targeting specificity in the RMTg is provided in Figure S1B of the supplementary materials. Utilizing the Hargreaves test, we observed that after chemogenetic inhibition of the LHb-RMTg excitatory pathway, the PWTL of the hM4D-saline group significantly increased to 4.02 ± 0.23 s. The mCherry group exhibited a PWTL of 4.06 ± 0.24 s, whereas the mCherry-saline group decreased to 3.2 ± 0.17 s. A significant increase in the hM4D-saline group was observed compared with that in the mCherry-saline group (Fig. 3D, F = 4.94, *p* = 0.017, *n* = 8 for mCherry group, *n* = 8 for mCherry-saline group, *n* = 8 for hM4D-saline group, one-way ANOVA, Tukey’s multiple comparison post hoc test). However, the Von Frey test yielded no significant variation in the PWMT among the compared groups. After chemogenetic inhibition of the LHb-RMTg excitatory pathway, the PWMT for the mCherry, mCherry-saline and hM4D-saline groups was 0.7 ± 0.08, 0.72 ± 0.16 and 0.7 ± 0.097 g, respectively (Fig. 3E, F = 0.013, *p* = 0.99, *n* = 8 for mCherry group, *n* = 8 for mCherry-saline group, *n* = 8 for hM4D-saline group, one-way ANOVA, Tukey’s multiple comparison post hoc test). These findings indicate that inhibition of the LHb-RMTg excitatory pathway reduces heat sensitivity without affecting the mechanical pain threshold. Additionally, the local effects induced by saline also enhanced pain sensitivity.

### Behavioral testing for pain in mice with CFA-induced inflammation following inhibiting the LHb-RMTg excitatory pathway

**Fig. 4:**
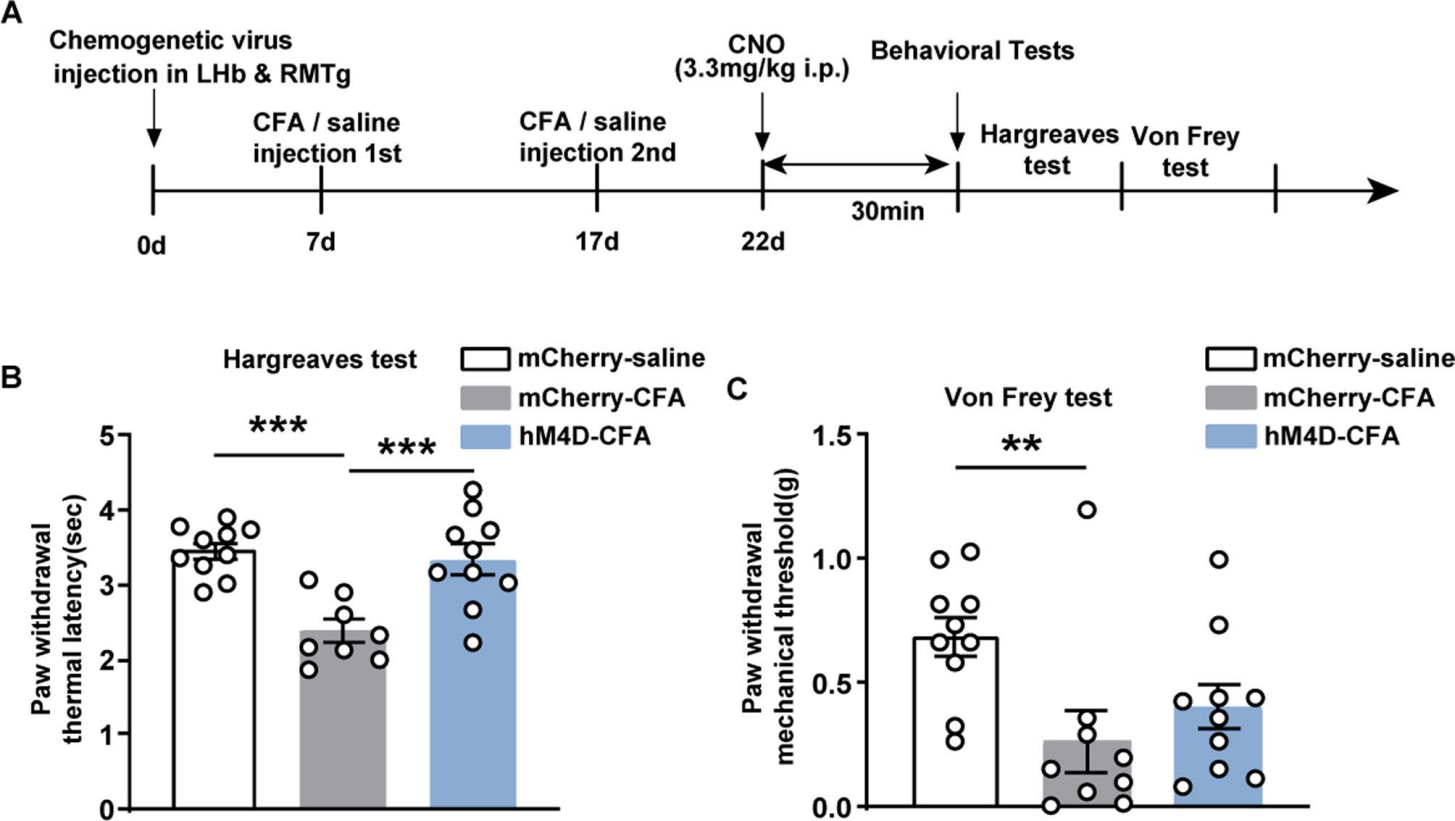
Behavioral testing for pain in mice with CFA-induced inflammation following inhibiting the LHb-RMTg excitatory pathway. (**A**) Flow diagram of the experiment for DREADD-mediated inhibition of the LHb-RMTg pathway in pain model. (**B**) Graphs of the Hargreaves test in the chemogenetic experiment. (**C**) Graphs of the Von Frey test in the chemogenetic experiment. Mean ± standard error of mean values are presented; **p < 0.01, ***p < 0.001. CFA, complete Freund’s adjuvant; DREADD, Designer Receptors Exclusively Activated by Designer Drug

We used a CFA-induced inflammatory pain to evaluate the impact of DREADD-mediated regulation of the LHb-RMTg circuit on pain thresholds in an inflammatory context. The experimental protocol is outlined in Fig. 4A. Similarly, AAV2/9-DIO-hM4D-mCherry or AAV2/9-DIO-mCherry serving as the control was delivered into LHb, while AAV2/Retro-CaMKIIa-Cre was distinctly delivered into RMTg. In the Hargreaves test, the PWTL of the mCherry-saline group was 3.46 ± 0.11 s. In contrast, the mCherry-CFA group exhibited a significantly reduced PWTL of 2.38 ± 0.15 s, indicating decreased heat sensitivity thresholds in mice with CFA-induced inflammation. Following the intraperitoneal administration of CNO to inhibit the LHb-RMTg pathway, the PWTL of the hM4D-CFA group increased to 3.34 ± 0.20 s, thereby suggesting that inhibition of the pathway reversed the decreased heat sensitivity thresholds recorded in the hM4D-CFA group compared with the mCherry-CFA group (Fig. 4B, F = 13.06, *p* = 0.0001, *n* = 10 for mCherry-saline group, *n* = 8 for mCherry-CFA group, *n* = 10 for hM4D-CFA group, one-way ANOVA, Tukey’s multiple comparison post hoc test). In the Von Frey test, the PWMT of the mCherry-saline group was 0.69 ± 0.08 g, whereas that of the mCherry-CFA group was significantly reduced (0.27 ± 0.12 g), reflecting a marked decrease in mechanical pain thresholds. However, following the intraperitoneal administration of CNO, the PWMT of the hM4D-CFA group remained at 0.4 ± 0.09 g. These findings show that inhibiting the LHb-RMTg pathway fails to restore the reduced mechanical threshold observed in the hM4D-CFA group compared to the mCherry-CFA group (Fig. 4C, *p* = 0.0093, *n* = 10 for mCherry-saline group, *n* = 9 for mCherry-CFA group, *n* = 10 for hM4D-CFA group, Kruskal–Wallis test, Dunn’s multiple comparison test).

### Pain behavioral testing following optogenetic Inhibition of the Vglut2^LHb − RMTg^ pathway

**Fig. 5:**
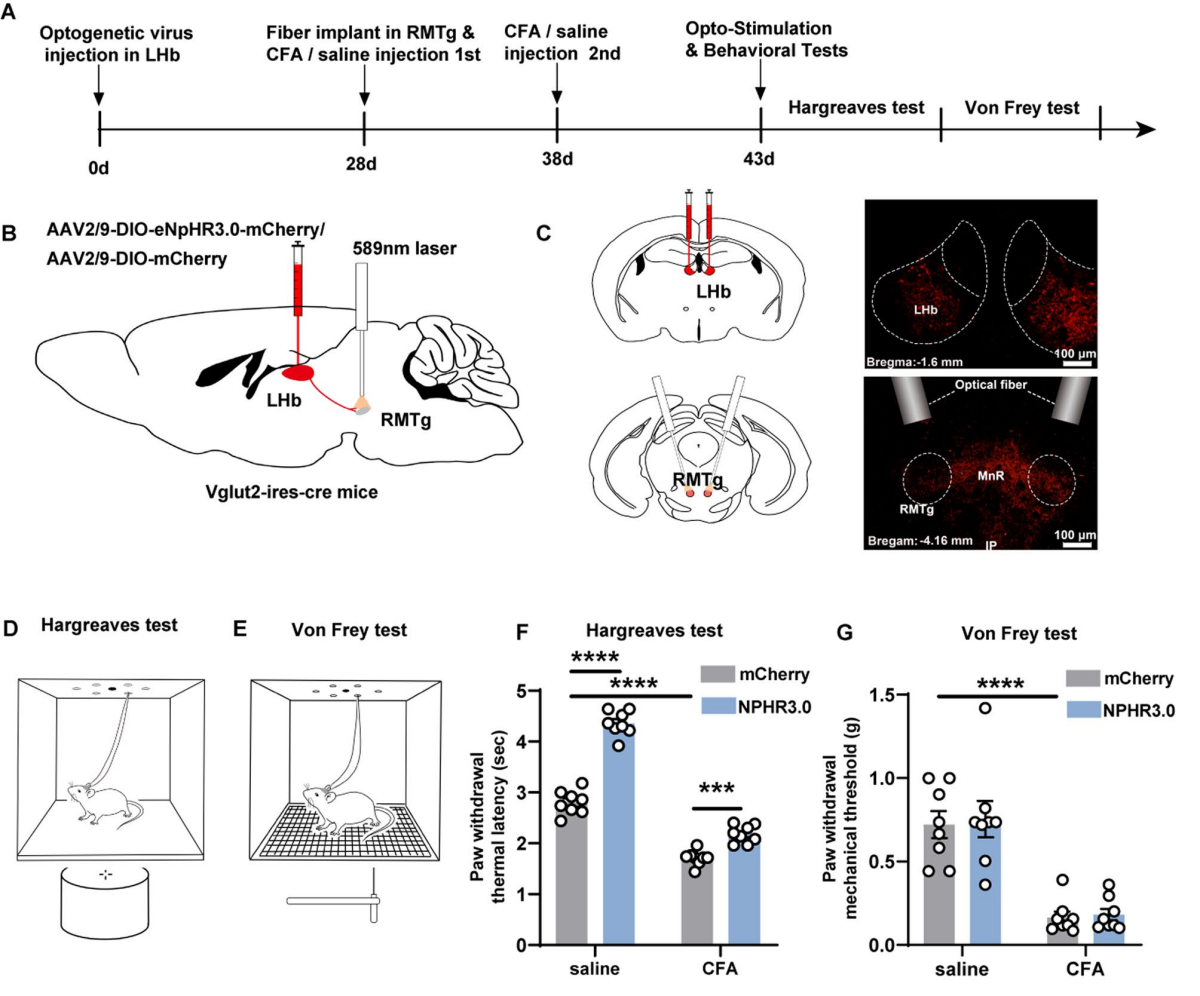
Pain behavioral testing following optogenetic inhibition of the Vglut2^LHb − RMTg^ pathway. (**A**) Flow diagram of the experiment for optogenetic inhibition of the LHb-RMTg pathway. (**B**) Schematics: AAV2/9-DIO-eNpHR3.0-mCherry or AAV2/9-DIO-mCherry is microinjected into LHb, and optical fiber is implanted above RMTg. (**C**) Fluorescence representative images: expression of AAV2/9-DIO-eNPHR3.0-mCherry in LHb and its terminals in RMTg (scale bar, 100 μm). (**D**, **F**) Schematics and corresponding graphs illustrating the Hargreaves test performed in the optogenetic experiment. (**E**, **G**) Schematics and corresponding graphs illustrating the Von Frey test performed in the optogenetic experiment. Mean ± standard error of mean values are presented; ***p < 0.001and ****p < 0.0001. LHb, lateral habenula; RMTg, rostromedial tegmental nucleus; MnR: Median raphe nucleus; IP: Interpeduncular nucleus

To further elucidate the instantaneous regulation of pain associated with glutamatergic terminals from LHb to RMTg, we conducted behavioral testing following optogenetic inhibition of the Vglut2^LHb − RMTg^ pathway (Fig. 5A). AAV encoding the light-sensitive chloride pump AAV2/9-DIO-eNPHR3.0-mCherry or AAV2/9-DIO-mCherry was selectively expressed in LHb of Vglut2-ires-cre mice (Fig. 5B). Representative expression of AAV2/9-DIO-eNPHR3.0-mCherry in LHb and its terminals in RMTg are displayed in Fig. 5C. Figure 5D and E show schematic representations of the Hargreaves and Von Frey tests, respectively. During the Hargreaves test, the PWTL was notably longer in the NPHR3.0-saline group (4.36 ± 0.085 s) than in the mCherry-saline group (2.8 ± 0.083 s) following optogenetic inhibition of the Vglut2^LHb − RMTg^ pathway. In contrast, when inflammation was induced with CFA, the PWTL of the mCherry-CFA group significantly decreased to 1.71 ± 0.051 s, indicating a drastic drop in heat sensitivity thresholds. Upon application of a 589-nm laser, the PWTL in the NPHR3.0-CFA group rose to 2.18 ± 0.06 s, suggesting a restoration of the CFA-induced decrease in heat sensitivity thresholds (Fig. 5F, F (1, 28) = 57.32, *p* < 0.0001, *n* = 8 for mCherry-saline group, *n* = 8 for mCherry-CFA group, *n* = 8 for NPHR3.0-saline group, *n* = 8 for NPHR3.0-CFA group, two-way ANOVA, post hoc Bonferroni’s tests). The Von Frey test, evaluating mechanical thresholds, showed no marked variation between the NPHR3.0-saline (0.75 ± 0.11 g) and mCherry-saline (0.72 ± 0.081 g) groups following optogenetic inhibition of the Vglut2^LHb − RMTg^ pathway. Similarly, when inflammation was induced with CFA, the mCherry-CFA group exhibited a significantly reduced PWMT of 0.17 ± 0.035 g compared with the mCherry-saline group. Following optogenetic inhibition in the NPHR3.0-CFA group, the PWMT remained unchanged at 0.18 ± 0.034 g, indicating that inhibiting the Vglut2^LHb − RMTg^ pathway had no significant influence on the mechanical thresholds (Fig. 5G, F (1, 28) = 0.012, *p* = 0.92, *n* = 8 for mCherry-saline group, *n* = 8 for mCherry-CFA group, *n* = 8 for NPHR3.0-saline group, *n* = 8 for NPHR3.0-CFA group, two-way ANOVA, post hoc Bonferroni’s tests).

## Discussion

This study identified a novel neural circuit involved in pain modulation. Our findings demonstrate that activation of the excitatory circuit from LHb to RMTg modulates nociception, potentially via its effect on the DRN. Aversive emotions typically show increased pain sensitivity [25]. LHb, an “anti-reward center,” is activated by aversive emotional cues, such as stress, fear, or negative reward [1, 2, 5]. RMTg, a major GABAergic nucleus, receives a negative signal from LHb [17]. This close morphological and functional relationship between LHb and RMTg neurons suggests that the LHb-RMTg circuit participates in pain regulation.

Our study demonstrated an excitatory pathway from glutamatergic neurons in LHb to RMTg neurons. Under normal conditions, activating RMTg-projecting LHb excitatory neurons with hM3D decreased the heat sensitivity thresholds. Conversely, deactivating the LHb-RMTg circuit via DREADD-mediated inhibition increased heat sensitivity thresholds. These findings establish a correlation between the LHb-RMTg circuit activity and heat sensitivity thresholds. Moreover, inhibiting RMTg-projecting LHb neurons reversed the decreased heat sensitivity thresholds under inflammatory pain conditions induced by CFA, suggesting that inflammatory pain is regulated by the LHb-RMTg circuit. Optogenetic inhibition of glutamatergic terminals from LHb to RMTg in Vglut2 mice increased heat sensitivity thresholds. Moreover, optogenetic inhibition of the Vglut2^LHb − RMTg^ pathway reversed the decrease in heat-sensitivity thresholds of the inflammation model. Our findings suggest that the excitatory pathway from LHb to RMTg maintains heat sensitivity.

Previous studies that manipulated RMTg GABAergic neurons [18] indicate that the RMTg contributes to nociceptive sensations potentially activated by LHb. In our study, the LHb-RMTg circuit modulates CFA-induced heat hypersensitivity. Further evidence suggests that peripheral inflammatory pain models, such as CFA-induced vulvodynia, drive multi-level neuroplasticity involving spinal sensitization and supraspinal reorganization, including motivational behaviors [26].Photogenetic studies have found that driving the LHb-RMTg circuit increases immobility in forced swimming tests and advances behavioral avoidance [19, 20], implying that the LHb-RMTg circuit integrates a variety of motivational behaviors related to pain. These results suggest that this circuit may mediate supraspinal reorganization induced by peripheral inflammatory pain.

Interestingly, we found that manipulation of the LHb-RMTg pathway exhibits high specificity in modulating thermal nociception. Notably, the LHb expresses transient receptor potential vanilloid type 1 (TRPV1), which is pivotal for thermal sensation [27, 28]. Furthermore, elevation of TRPV1 function mediates hyper-glutamatergic transmission and hyperactivity of LHb neurons, while inhibition of LHb TRPV1 reduces thermal hyperalgesia in ethanol-withdrawn rats [29]. However, whether the LHb-RMTg circuit engages TRPV1-dependent mechanisms to regulate thermal nociception remains further explored. Additionally, there may exist other neurobiological mechanisms, which could explain the preference for processing of thermal over mechanical nociceptive stimuli.

Immunohistochemistry has shown that the quantity of c-Fos–labeled cells in the DRN declined after DREADD-mediated activation of the LHb-RMTg pathway, thereby suggesting that LHb inhibits the DRN via the indirect brain area of RMTg [16, 30, 31]. Stimulation of the DRN reportedly has important antinociceptive effects [14], and lesioning of the DRN inhibits morphine-induced analgesia [32]. The DRN also inhibits pain sensitivity by releasing 5-HT [4]. These studies indicate that increasing the activity of the LHb-RMTg circuit might result in the inhibition of the raphe serotonergic system, contributing to pain sensitivity. The LHb-RMTg-DRN pathway requires further study to examine whether the serotoninergic neurons in the DRN may be regulated by GABAergic neurons in the RMTg, which receives projections from the LHb. Functionally, the LHb-RMTg-DRN pathway may be involved in pain regulation, pain-induced sleep disorders, and pain-induced depression.

## Conclusion

These results provide behavioral indication that the excitatory LHb-RMTg circuit is necessary for maintaining heat sensitivity, suggesting that the potential underlying mechanism may involve inhibiting 5-HT neurons in the DRN. This study suggests a novel circuit for pain generation and identifies new targets for the prevention and treatment of inflammatory pain.

## Electronic supplementary material

Below is the link to the electronic supplementary material.

Supplementary Material 1

## Data Availability

No datasets were generated or analysed during the current study.

## Abbreviations

DRN: Dorsal raphe nucleus
LHb: Lateral habenula
RMTg: Rostromedial tegmental nucleus
5-HT: 5-hydroxytryptamine
DREADD: Designer Receptors Exclusively Activated by Designer Drugs
CFA: Complete Freund’s adjuvant
CNO: Clozapine-N-oxide
AP: Anteroposterior
ML: Mediolateral
DV: Dorsoventral
PWTL: Paw withdrawal thermal latency
PWMT: Paw withdrawal mechanical threshold
ANOVA: Analysis of variance
MnR: Median raphe nucleus
IP: Interpeduncular nucleus
TRPV1: Transient receptor potential vanilloid type 1
CTB-488: Alexa Fluor 488-conjugated cholera toxin subunit B
