# Supplementary material for: Lateral habenula-rostromedial tegmental nucleus circuit mediates inflammatory pain in mice
Source: J Headache Pain. 2025 May 6;26(1):105. doi: 10.1186/s10194-025-02052-w (PMC12057000; doi:10.1186/s10194-025-02052-w)
Supplement: Supplementary file 1 — Supplementary Material 1 [file 10194_2025_2052_MOESM1_ESM.docx]

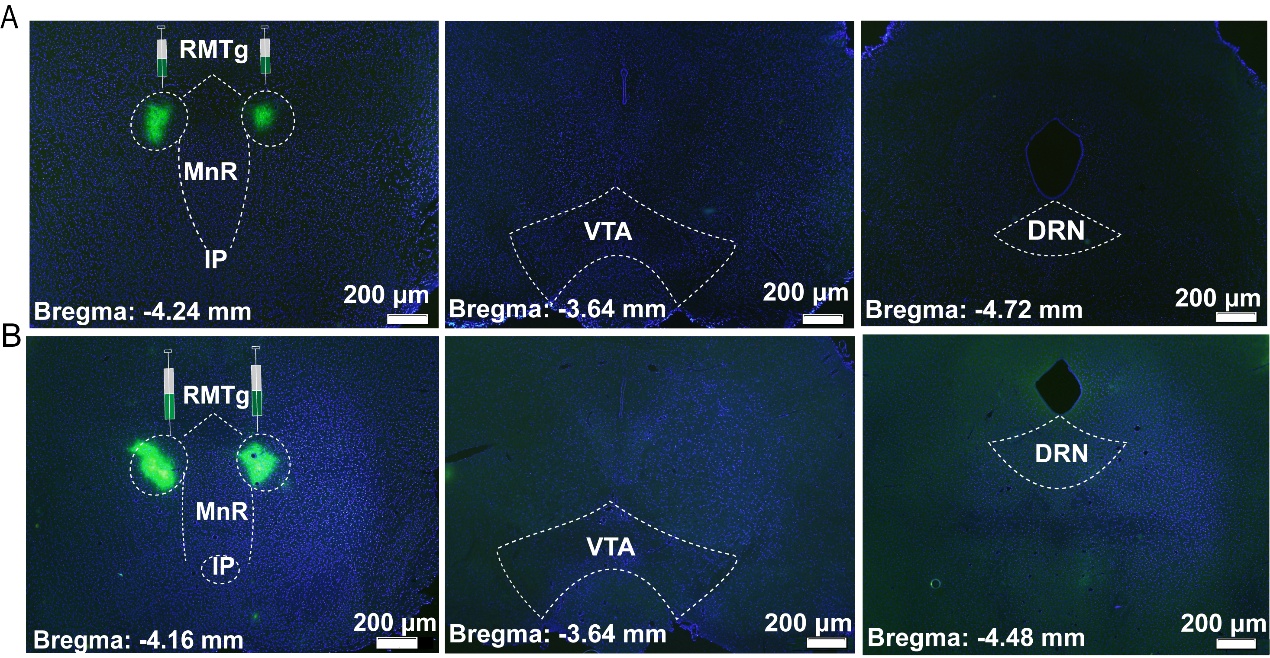


**Figure S1.** **Validation of viral** **targeting specificity in the rostromedial tegmental nucleus (RMTg) for chemogenetic activation and inhibition experiments.**

1. For chemogenetic activation experiments. Co-injection of AAV2/Retro-CaMKIIα-Cre and Alexa Fluor 488-conjugated cholera toxin subunit B (CTB-488) into the RMTg. Histological analysis demonstrated RMTg-specific targeting with no off-target signals in adjacent regions including the median raphe nucleus (MnR), ventral tegmental area (VTA) or dorsal raphe nucleus (DRN). (B) For chemogenetic inhibition experiments. Fluorescence imaging similarly demonstrated RMTg- specific targeting with no detectable signals in the MnR, VTA or DRN.
